# Long-term outcomes of surveillance or endoscopic therapy for low-grade dysplastic Barrett’s according to a selective management algorithm

**DOI:** 10.1055/a-2778-3907

**Published:** 2026-02-27

**Authors:** Tony He, Mark Lai, Kiran G Iyer, Sara Vogrin, John L Slavin, Edward H Tsoi, Bronte Holt, Paul Desmond, Andrew CF Taylor

**Affiliations:** 1 60078 Gastroenterology, St Vincent's Hospital Melbourne Pty Ltd., Fitzroy, Australia; 2 85084 Faculty of Medicine Dentistry and Health Sciences, The University of Melbourne, Melbourne, Australia; 3 85084 Statistics, Faculty of Medicine Dentistry and Health Sciences, The University of Melbourne, Melbourne, Australia; 4 60078 Pathology, St Vincent's Hospital Melbourne Pty Ltd., Fitzroy, Australia; 5 60078 Center for Interventional Endoscopy, St Vincent's Hospital Melbourne Pty Ltd., Fitzroy, Australia; 6 85084 Medicine, Faculty of Medicine Dentistry and Health Sciences, The University of Melbourne, Melbourne, Australia

**Keywords:** Endoscopy Upper GI Tract, Barrett's and adenocarcinoma, Reflux disease, Endoscopic resection (ESD, EMRc, ...), RFA and ablative methods

## Abstract

**Background and study aims:**

Current European and American guidelines conflict in their recommendations for surveillance versus endoscopic therapy for low-grade dysplastic Barrett’s (LGD). We aimed to evaluate the performance of a selective management algorithm and provide real-world outcomes.

**Patients and methods:**

Data on 497 patients with dysplastic Barrett’s were collected prospectively between 2008 and 2022 at a Barrett’s referral unit. LGD was defined as confirmation of LGD by an expert gastrointestinal pathologist. Persistent unifocal LGD or multifocal LGD were considered high-risk features for progression and patients underwent endoscopic eradication therapy (EET). Patients with non-persistent unifocal LGD were deemed low-risk and were surveilled. Primary outcome was progression rate to high grade dysplasia or neoplasia.

**Results:**

A total of 135 patients had LGD (median [interquartile range] follow up: 4.8 years [1.0–7.1]): 22 patients met low-risk criteria and were surveilled (LR-S), eight patients met high-risk criteria and were surveilled (HR-S; patient preference n = 4, medical comorbidities n = 4), and 105 patients met high-risk criteria and underwent EET (HR-EET). Progression rates were similar between the LR-S and HR-EET cohorts (4.5% [n = 1/22] vs. 6.7% [n = 8/105]; *P* = 0.43). The HR-S group had a significantly higher progression rate (25% [n = 2/8]; *P* = 0.04). Univariable analysis showed reflux esophagitis (sub-distribution hazard ratio 3.21, 95% confidence interval 1.02–10.1, *P* = 0.04) was associated with progression risk in the high-risk LGD cohort only.

**Conclusions:**

This selective management algorithm for LGD is safe. Surveillance is appropriate in low-risk LGD patients. Patients with high-risk features who are surveilled and/or have reflux esophagitis may have an increased progression risk and should undergo EET with optimized acid suppression therapy.

## Introduction

Barrett’s esophagus (BE) with low-grade dysplasia (LGD) is a premalignant condition which increases risk of esophageal adenocarcinoma (EAC) 1 . EAC is associated with a poor prognosis, with an incidence in the Western world that continues to rise 2 .

There are substantial data supporting endoscopic eradication therapy (EET) for patients with HGD and T1a EAC; however, the necessity for endoscopic treatment of LGD remains controversial 3 4 5 . This stems from three factors: 1) widely variable published rates of progression of 0.4% to 13.4% per patient per year; 2) high interobserver variability of LGD diagnosis among pathologists; and 3) acknowledgement that there are different risk-phenotypes of LGD, but relatively limited data that risk stratify LGD patients into appropriate surveillance versus treatment groups 6 7 8 9 . Therefore, it is unsurprising that current European and American guidelines conflict in their LGD management recommendations 3 4 5 . There is universal agreement that diagnosis of LGD should be confirmed by an expert gastrointestinal pathologist, with follow-up endoscopy performed in 6 months to assess for persistent LGD. However, if persistent LGD is identified, American Society for Gastrointestinal Endoscopy guidelines suggest that either surveillance or EET are acceptable approaches whereas both the European Society of Gastrointestinal Endoscopy (ESGE) and British Society of Gastroenterology guidelines recommend EET.

The argument favoring EET is largely driven by Phoa and Pouw’s respective randomized controlled trials (RCTs) 10 11 . Both trials reported that EET markedly reduced risk of progression of LGD to HGD and EAC. In a subsequent RCT with a similar design, Barret et al reported on a French cohort of 82 patients (40 patients in the radiofrequency ablation (RFA) and 42 patients in the surveillance group) 12 . Barret et al also detected a modest reduction in progression in the RFA cohort (12.5% vs 26.2%, *P* = 0.15); however, the findings are limited by the small sample size and unconventional primary outcome: prevalence of LGD at 3 years. However, despite these three RCTs, it remains unclear whether the likely modest benefits of EET outweigh both the procedure risk and cost in the real world.

Furthermore, none of the three RCTs assess the impact of risk-stratification tools in clinical decision making. Reliance on dysplasia degree alone continues to be a critical barrier to risk-based management of LGD. Currently, confirmation of LGD by an expert gastrointestinal pathologist, persistent LGD (LGD detected on more than one endoscopy), multifocal LGD, and nodularity at index endoscopy have been reported as risk factors for progression 10 11 12 13 14 15 16 . Ultimately, a selective algorithm that highlights high-risk versus low-risk LGD patients may maximize benefits of EET and reduce procedure risk and cost to patients who do not require EET and can safely be surveilled.

Thus, the aim of this study was to evaluate performance of a selective management algorithm and: 1) determine rates of progression; 2) determine the rate of adverse events; 3) identify possible predictive factors for progression; and 4) provide overall long-term outcomes of patients with LGD.

## Patients and methods

### Study design

We conducted a retrospective cohort study of adult patients (18 years or older) referred to a state-wide Barrett’s referral unit (BRU) with dysplastic BE or T1a EAC in Victoria, Australia. Data were collected prospectively and maintained in a database between November 2008 to November 2022. Minimum follow-up time for inclusion in the study was 12 months. This study was approved by the St Vincent’s Hospital Human Research Ethics Committee (HREC-D 161/09).

### Study definitions

LGD was defined as confirmation of LGD by an expert gastrointestinal pathologist. Unifocal LGD was defined as LGD present at one biopsy level within a BE segment. Multifocal LGD was defined as LGD present at more than one biopsy level within a BE segment. Persistent LGD was defined as LGD confirmed at subsequent surveillance endoscopy at the same or different level. Presence of reflux esophagitis was defined as any grade of reflux esophagitis (Los Angeles Classification 17 ) despite proton pump inhibitor (PPI) therapy at the initial BRU assessment endoscopy.

### Lesion assessment protocol

Referral histology of all patients was reviewed by an expert gastrointestinal pathologist at a multidisciplinary meeting to confirm the histological diagnosis. Subsequently, endoscopic assessment was performed at our center by an accredited gastroenterologist with prior advanced therapeutic endoscopy training in a recognized fellowship program. All procedures were performed using a high-definition gastroscope (GIF-HQ190 or HQ180, Olympus Australia) and examination performed with high-definition white light endoscopy and narrow band imaging with dual image magnification and a transparent cap for image stabilization. Barrett’s length was documented using the Prague classification 18 . Targeted biopsies or endoscopic resection of visible lesions suspicious for dysplasia or neoplasia were taken prior to surveillance biopsies. Surveillance biopsies were taken as per the Seattle protocol. Internal histology was then reviewed by an expert gastrointestinal pathologist and often re-discussed at a multidisciplinary meeting to confirm histological diagnosis.

### Selective management algorithm

For patients with expert gastrointestinal pathologist-confirmed LGD, EET was offered to patients who were deemed high risk for progression. High-risk features included: 1) persistent unifocal LGD (LGD detected on two or more endoscopic procedures at our center with confirmation by an expert gastrointestinal pathologist; or 2) multifocal LGD. For patients who underwent EET, endoscopic resection was performed for all visible lesions with proven or suspected dysplasia by endoscopic resection, followed by RFA of residual flat BE. In patients with non-visible dysplasia and flat BE, RFA was performed.

Surveillance was offered to patients with low-risk features for progression. Low-risk features included non-persistent unifocal LGD (LGD detected only once at our center) with flat, regular BE mucosal and vascular pattern. Surveillance protocol was a surveillance endoscopy performed at 6 months with Seattle protocol biopsies, followed by annual endoscopy. After two consecutive negative endoscopies for dysplasia, standard surveillance for patients with non-dysplastic BE was initiated.

All patients were prescribed long-term high-dose PPIs, irrespective of whether they underwent surveillance or were in the EET cohort. If persistent reflux was noted on endoscopic examination, patients had acid suppression optimization with additional histamine 2 receptor (H2R) antagonists and/or sucralfate.

### Post CRIM surveillance protocol

For participants who underwent EET and achieved complete remission of intestinal metaplasia (CRIM), surveillance endoscopy was performed at 3, 6, 9 and 12 months thereafter. If any dysplastic recurrence (LGD, HGD) or early neoplasia was detected, EET was performed (endoscopic resection for visible lesions and RFA for non-visible, flat lesions) until CRIM was reachieved.

### Study outcomes and statistical analysis

The primary study outcome was the rate of LGD progression to HGD or EAC. Secondary outcomes were progression timeline, progression histology, procedure-related adverse events (AEs), long-term outcomes, predictors of progression and rates of histological upstaging or downstaging after expert gastrointestinal pathologist review. Data were summarized as mean (± standard deviation), median (interquartile range [IQR]) or proportions (%), as warranted. Cumulative incidence of progression in the presence of competing risk (death) was estimated using the method described by Coviello et al 19 . Kaplan-Meier estimates are also presented. Univariable analysis using a competing risks regression model (Fine and Gray method) was performed to identify factors associated with progression. All statistical analysis was performed using STATA Version 17.0 (StataCorp LLC).

## Results

### Patient cohort

A total of 497 patients with suspected dysplastic BE or T1a EAC were referred and assessed at our center between November 2008 and November 2022. Within this cohort, 165 patients (33.2%) were referred with LGD, 190 patients (38.2%) with HGD and 142 patients (28.6%) with suspected T1a EAC.

After expert gastrointestinal pathologist review and expert endoscopic BRU assessment, 135 patients were confirmed to have true LGD and were included in the final analysis. Characteristics of patient demographics, BE features, and treatment details are summarized in Table 1 . Median (IQR) follow-up time was 4.1 years (1.0, 7.1).

**Table:** **Table 1** Characteristics of patients with confirmed LGD (n = 135).

Variable	Value
Age, years, median (IQR)	67.9 (61.9- 75.0)
Male (%)	77.6%
Length of BE (Prague C in cm), median (IQR)	C2 (0–5)
Length of BE (Prague M in cm), median (IQR)	M4 (2–7)
Hiatal hernia present (%)	95.5%
Presence of reflux esophagitis (n, %)	30 (22.4%)
Persistent LGD (n, %)	66 (49.3%)
Unifocal LGD (n, %)	64 (47.4%)
Multifocal LGD (n, %)	71 (52.6%)
Visible lesions (n, %)	68 (50.4%)
Surveillance	30 (22.4%)
Treatment: EMR and RFA (n, %)	39 (28.9%)
Treatment: RFA monotherapy (n, %)	51 (38.1%)
Treatment: EMR monotherapy (n, %)	15 (11.2%)
*Data presented as number (%), mean (+/- standard deviation), or median (IQR). BE, Barrett’s esophagus; EMR, endoscopic mucosal resection; IQR, interquartile range; LGD, low-grade dysplasia; RFA, radiofrequency ablation.	

### Expert gastrointestinal pathologist review

**Fig. 1:**
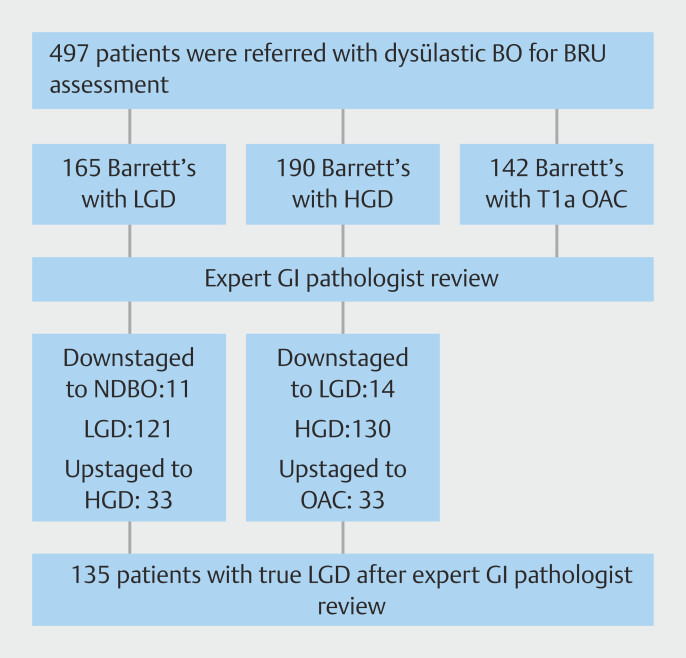
Patient flow chart.

Of the 165 patients referred with LGD, 121 (73.3%) had confirmed LGD. Of the remaining 44 patients, 33 (20%) were upstaged to T1a EAC (n = 8; 4.8%) or HGD (n = 25; 15.2%) and the remaining 11 patients (6.7%) were downstaged to either indefinite for dysplasia (n = 3; 1.8%) or non-dysplastic BE (n = 8; 4.8%). Of the 190 patients referred with HGD, 14 (7.4%) were downstaged to LGD. Of the remaining 176 patients, 130 (68.4%) had confirmed HGD and 46 (24.2%) were upstaged to T1a EAC. Fig. 1 shows the patient flow chart.

### Selective management algorithm outcomes

#### Surveillance for low-risk LGD (LR-S cohort)

In our cohort, 22 patients (16.3%) were deemed to have low-risk features (unifocal, non-persistent LGD) and were surveilled. Median (IQR) follow-up time was 4.4 years (0.9, 7.8) ( Table 2 ).

**Table:** **Table 2** Surveillance versus EET patient characteristics.*

Variable	Low-risk LGD: surveillance (n = 22)	High-risk LGD: surveillance (n = 8)	High-risk LGD: EET (n = 105)	*P* value
Age, years, median (IQR)	67.7 (60.3, 75)	71.4 (64.3, 80)	68.5 (63.2, 77)	0.825
Male (%)	72.7%	75%	81.9%	0.468
Referral histology (n, %):				0.047
LGD	22 (100%)	7 (87.5%)	92 (87.6%)	
HGD	0 (0%)	1 (12.5%)	13 (12.4%)	
Length of BE (Prague C in cm), median (IQR)	3 (0, 7)	2 (0, 4)	2 (0, 4)	0.316
Length of BE (Prague M in cm), median (IQR)	5 (2, 8)	4 (1, 7)	4 (2, 6)	0.731
Hiatus hernia present (%)	95.4%	87.5%	96.2%	0.973
Presence of reflux esophagitis (n, %)	3 (13.6%)	2 (25%)	25 (23.8%)	0.157
Persistent LGD (n, %)	0 (0.0%)	4 (50%)	58 (55.2%)	0.050
Unifocal LGD (n, %)	22 (100.0%)	4 (50%)	38 (36.2%)	0.067
Multifocal LGD (n, %)	0 (0.0%)	4 (50%)	67 (63.8%)	0.045
Visible lesions (n, %)	0 (0.0%)	2 (25%)	65 (61.9%)	0.036
*Data presented as number (%), mean (+/- standard deviation), or median (IQR). BE, Barrett’s esophagus; IQR, interquartile range; HGD, high-grade dysplasia; LGD, low-grade dysplasia; SD, standard deviation.				

Of the 22 patients who were deemed low risk and surveilled, one patient progressed to T1a EAC (4.5%). The patient who progressed to T1a EAC (baseline C1M4 BE) had been referred back to the community for surveillance and was lost to follow up for 2 years (time to progression 3 years). The patient underwent successful endoscopic resection (EMR), followed by RFA and has achieved complete eradication of IM (maintained at last follow up).

There were no procedure-related AEs documented in patients who underwent surveillance.

#### Surveillance for high-risk LGD (HR-S cohort)

Eight patients (5.9%) who were deemed high risk (persistent unifocal LGD n = 4 and multifocal LGD n = 4) underwent surveillance due to either patient preference (n = 4) or significant medical comorbidities precluding treatment (n = 4). Median (IQR) follow-up time was 3.6 years (0.8, 6.5) ( Table 2 ).

Of these eight patients, two (25%) progressed to HGD (n = 1) and T1b EAC (n = 1). Median (IQR) time to progression was 2.6 years (1.3–6.5). The patient with T1b EAC (baseline C4M5 BE) was deemed to be fit for an esophagectomy and underwent surgical resection (final histology: high risk T1bN0M0). The patient with HGD (baseline C2M3 BE) has achieved complete eradication of BE after undergoing EMR, followed by RFA therapy.

During the surveillance phase, no procedure-related AEs occurred.

#### Endoscopic eradication therapy for high-risk LGD (HR-EET cohort)

One hundred and five patients (77.8%) were deemed to have high-risk features (multifocal LGD n = 67, persistent unifocal LGD n = 38) that required EET (51 underwent RFA monotherapy). Median (IQR) follow-up time was 4.3 years (1.1, 7.3) ( Table 2 ).

CRIM was achieved in 82 patients (78.1%) and 23 patients were still undergoing endoscopic therapy at last follow up (12 of 23 patients had achieved complete remission from dysplasia [CRD]).

Eight patients (7.6%) progressed to HGD (n = 6) and T1a EAC (n = 2). No patients were lost to follow up and all were detected at our center. Median (IQR) time to progression was 3.3 years (1.6–7.1).

Of the eight patients who progressed, six had recurrences (HGD n = 5, T1a EAC n = 1) after initial successful EET (i.e., post achievement of complete eradication of IM (CRIM)). The two patients who progressed to T1a EAC had baseline C9M11 and C3M6 BE segments, respectively. All eight patients underwent further EET and the majority had achieved CRIM at last follow up (n = 5). The remaining three patients have achieved CRD only and are still undergoing additional EET.

Procedure-related AEs included: stricture formation n = 4, 3.8%), post-procedure chest pain (n = 8, 8.6%), bleeding (n = 3, 2.9%), and perforation (n = 1, 0.95%).

### Overall long-term outcomes of LGD

Of the 135 patients with confirmed LGD, a total of 11 patients (8.2%) progressed to HGD (n = 7), T1a EAC (n = 3) and T1b EAC (n = 1). Ten of 11 patients (90.9%) who progressed were successfully treated endoscopically, one patient required an esophagectomy. None of the patients who were downstaged from LGD to non-dysplastic BE (NDBE) progressed to advanced dysplasia or neoplasia. One of the 14 patients who were downstaged from HGD to LGD progressed to HGD (developed post EET).

Overall, cumulative incidence of progression at 2 years (considering competing risk of dying) was 3.8% (95% confidence interval [CI] 1.2–8.8) and an additional 10.5% (95% CI 6.3–25.4) at 8 years ( **Supplementary Fig. 1** ). The estimated annual incidence progression rate was 2.86% per patient per year of follow up (95% CI 1.4–11.2). **Supplementary Fig. 2** shows Kaplan-Meier estimates of cumulative incidence with 95% CIs for progression by year. This pattern was similar when stratified by surveillance versus EET ( **Supplementary Fig. 3** ).

### Predictors of progression

Univariable analysis (Fine and Gray method) was performed to identify demographic, endoscopic, and treatment factors that may be associated with progression in patients with low- and high-risk LGD. In the high-risk LGD cohort, gastroesophageal reflux esophagitis was associated with a 3-fold increased risk of progression to HGD or EAC (sub-hazard ratio [SHR] 3.21, 95% CI 1.02–10.11, *P* = 0.04). In the low-risk LGD cohort, no factors associated with progression were detected. Male gender could not be quantified as no females progressed ( Table 3 ).

**Table:** **Table 3** Univariable analysis for predictors of progression to HGD or EAC in low-risk and high-risk LGD cohorts.

Variable	Low-risk cohort (n = 22): Sub-hazard ratio (SHR), 95% CI	*P* value	High-risk cohort (n = 113): Sub-hazard ratio (SHR), 95% CI	*P* value
Age	0.98 (0.92–1.06)	0.377	1.05 (0.96–1.14)	0.286
Male sex	N/A	N/A	N/A	N/A
Referral histology: LGD	N/A	N/A	Reference	
Referral histology: HGD	N/A	N/A	2.73 (0.61–12.32)	0.191
Presence of hiatal hernia	0.33 (0.09–2.15)	0.668	0.24 (0.03–1.81)	0.165
Presence of reflux esophagitis	1.03 (0.90–1.30)	0.277	3.21 (1.02–10.11)	0.046
Length of BE, Prague C	0.99 (0.88–1.15)	0.778	1.01 (0.91–1.13)	0.825
Length of BE, Prague M	0.95 (0.86–1.09)	0.331	1.07 (0.95–1.20)	0.261
Persistent LGD	N/A	N/A	0.84 (0.26–2.68)	0.768
Unifocal LGD	Reference		Reference	
Multifocal LGD	N/A	N/A	0.53 (0.16–1.76)	0.302
Visible lesions	N/A	N/A	1.32 (0.4–4.35)	0.645
Nodular lesion (Paris 0-IIa or Paris 0-Is)			1.88 (0.43–8.25)	0.404
Flat lesion with abnormal mucosal pattern (Paris 0-IIb)			1.02 (0.25–4.19)	0.973
BE, Barrett’s esophagus; CI, confidence interval; EAC, esophageal adenocarcinoma HGD, high-grade dysplasia; LGD, low-grade dysplasia.				

## Discussion

We demonstrate that our selective management algorithm for patients with low-grade dysplastic Barrett’s is safe, feasible, and efficacious. Our center’s approach safely identified low-risk patients appropriate for surveillance (LR-S progression 4.5%, n = 1 of 22 vs. HR-EET 6.7%, n = 8 of 105) and also recognized high-risk patients inappropriate for surveillance (HR-S progression 25%, n = 2 of 8). For patients that underwent surveillance, risk of EET-related AEs was mitigated (surveillance: 0% vs. EET: 15.2%) and treatment-related cost was reduced. When progression was detected, the majority (90.9%, n = 10 of 11) were successfully treated endoscopically and only one patient required an esophagectomy. Ultimately, our selective management algorithm is a safe and simple method of optimizing LGD care and supports the recent ESGE published guideline on LGD management.

The current argument favoring EET for LGD is largely driven by Phoa and Pouw’s respective RCTs 10 11 . The reported rate of progression in the surveillance arm was concerningly high at 33.8%. This rate, however, is far greater than any other published cohort, including our study (10%: LR-S n = 1 of 22 and HR-S n = 2 of 8). More recently in 2021, Barret et al provided additional RCT data (RFA n = 40 vs surveillance n = 42) 12 . There was a modest reduction in progression detected in the RFA cohort (12.5% vs 26.2%, *P* = 0.15); however, Barrett et al’s findings are limited by a small sample size and were also not statistically significant. Collectively, current RCT data on whether EET for LGD truly outweighs treatment-related risk and cost in the real world remain unclear. Unlike HGD or EAC, LGD management requires a nuanced approach. The widely variable progression rates and treatment benefit reported in the literature coupled with the challenges anatomical pathologists face in diagnosing LGD underline the wide spectrum of LGD phenotypic risk 6 7 8 9 10 11 12 20 . Our center devised a selective management algorithm to address this. Our treatment protocol delineated low- and high-risk LGD based on published risk factors, guidelines recommendations, and local expertise 3 4 5 6 7 8 9 10 11 12 13 14 15 16 20 . Low-risk LGD patients were surveilled and our progression rate was far lower than those reported by Phoa, Pouw, and Barrett’s RCTs (4.5% vs. 33.8% [Phoa, Pouw et al] vs 12.5% [Barrett et al]). Interestingly, in patients identified with high-risk LGD who were surveilled (patient preference or medical comorbidities limiting treatment), progression rates were similar to Phoa, Pouw, and Barrett et al’s cohorts (25% vs. 33.8% vs. 26.2%, respectively). In high-risk LGD patients who underwent treatment, progression was, as expected, relatively low (6.7%) and comparable with published RCT data. However, it is important to highlight that progression manifested predominantly as post-CRIM recurrence in our cohort (75%, n = 6 of 8). This finding underlines the crucial role of careful ongoing surveillance in LGD patients despite having undergone EET. When compared with Moole and Curvers et al’s retrospective data, our overall progression rate was similar (8.1% vs. 10.4% [Moole et al] vs. 13.4% [Curvers et al]) 15 21 . In summary, we propose that unlike HGD or EAC, there is a spectrum of disease risk for LGD. The high progression rates reported by Phoa, Pouw, and Barrett et al in the surveillance arm is representative of this. If high-risk patients are surveilled, they have a high risk of progression. Therefore, a selective management algorithm is better suited for LGD management.

Endoscopic therapy also carries treatment-related risk. In the aforementioned RCT data, treatment-related AEs were not insignificant with 16.9% to 19.1% of patients developing post-RFA complications 10 11 12 . In our cohort, the EET cohort had significantly more procedure-related AEs compared with our surveillance cohort (15.2% vs. 0%, *P* < 0.01). This is an important consideration when risk of progression is low for low-risk LGD (4.5%). Furthermore, despite the high rate of progression reported by Pouw and Phoa et al, there was no mortality difference when comparing the two cohorts and only one patient required an esophagectomy. Similarly, of our 11 patients who progressed, the majority (90.9%) were successfully treated endoscopically and did not require an esophagectomy and/or chemoradiation. Ultimately, this reaffirms that in the real world, treatment-related risk and cost may outweigh potential benefit of a universal EET approach for LGD. However, a good management algorithm for patients with LGD requires accurate risk stratification. Molecular and imaging biomarker studies have been explored as additional avenues of risk-stratifying patients 22 23 . In our study, we assessed patient, endoscopic, and treatment factors in both our low- and high-risk LGD patients. We detected that in our high-risk cohort, reflux esophagitis was associated with a three-fold increase in progression (SHR 3.21; *P* = 0.046). This is perhaps not a surprising finding, given that long-term, uncontrolled reflux esophagitis is a well-established risk factor for dysplastic BE 24 . Optimization of acid suppression therapy with PPI, H2R antagonists and/or sucralfate should always be prioritized in patients with dysplastic BE to reduce risk of progression.

It is relevant to note that in our cohort, after expert gastrointestinal pathologist review, only 6.6% of patients referred with LGD were downstaged to nondysplastic BE (NDBE). The rate of histological downstaging in our study was significantly lower than the 85% reported by Curvers et al 15 . Our center, however, is one of only two state-wide hospitals that offer and provide RFA therapy. The majority of our referrals are from tertiary hospitals with an expert gastrointestinal pathology service. This likely resulted in the lower discrepancy in LGD diagnosis. Furthermore, no patients who were downstaged to NDBE progressed to HGD or EAC.

In summary, our study provides real-world data to support a selective management algorithm approach to LGD. Our study, however, is not without limitations. This is a single-center retrospective study that was performed in an expert Barrett’s center. These data, therefore, are not generalizable to community hospitals without BE expertise (endoscopy and pathology). Furthermore, although follow-up times were relatively long in this study, it is still possible that recurrence risk may differ with longer follow-up times, especially given that median time to progression was 2.9 years and our median follow-up time was 4.1 years. In addition, a larger sample size would have greater power to detect changes in recurrence rate over time and identify other potential predictors of neoplastic progression (accounting for confounding factors; univariable analysis, rather than multivariable analysis. was performed, given the limited number of events).

## Conclusions

Our center’s selective management algorithm for LGD is safe, feasible, and may mitigate AEs associated with EET. Surveillance is appropriate in low-risk LGD patients. However, patients with high-risk features who are surveilled and/or have active reflux esophagitis may have an increased risk of progression and, thus, should undergo EET with optimized acid suppression therapy. Further prospective studies are required to validate this algorithm.

## Supplementary Material

Supplementary Material Zusatzmaterial
