# Supplementary material for: Long-term outcomes of surveillance or endoscopic therapy for low-grade dysplastic Barrett’s according to a selective management algorithm
Source: Endosc Int Open. 2026 Feb 27;14:a27783907. doi: 10.1055/a-2778-3907 (PMC12951032; doi:10.1055/a-2778-3907)

Thieme

Supplementary figure 1. Cumulative incidence of progression estimated using the Fine and Gray method in patients with LGD.

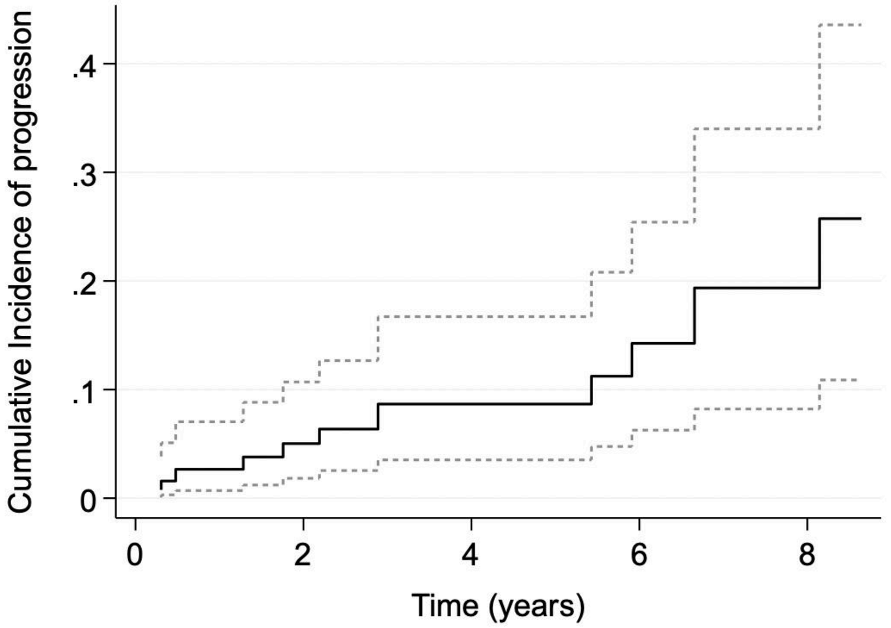

Supplementary figure 2. Kaplan Meier estimates of LGD progression to HGD or OAC.

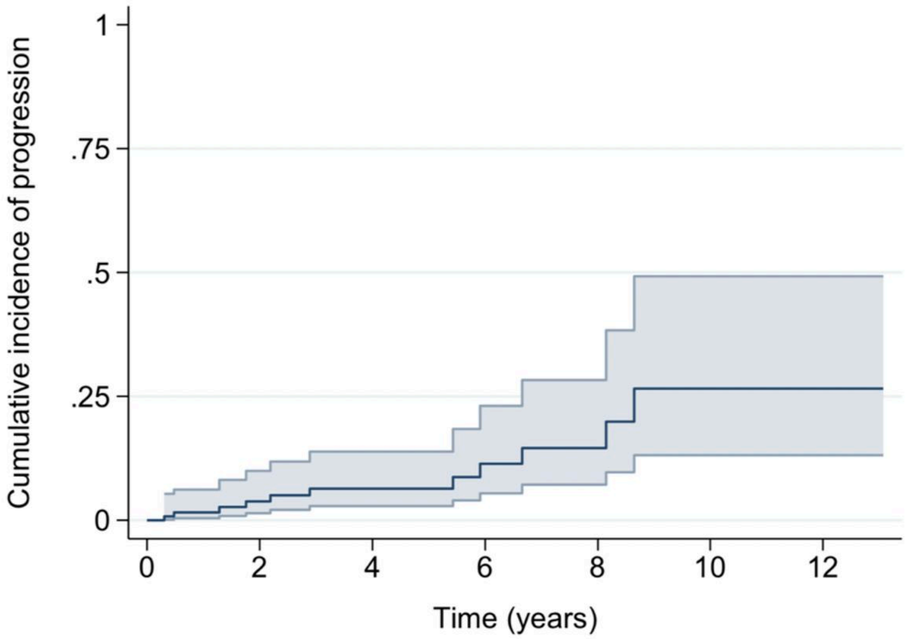

Supplementary figure 3. Kaplan Meier estimates of LGD progression to HGD or OAC, stratified by surveillance versus EET.

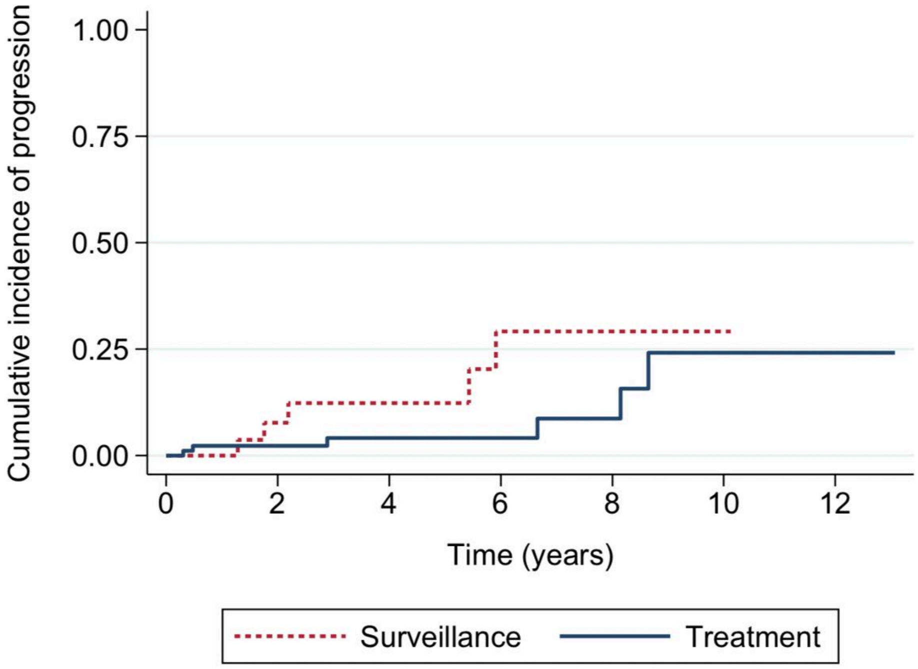

Supplement: Supplementary file 1 — Supplementary Material [file 10-1055-a-2778-3907_27947474.pdf]
